# Therapeutic recommendations and seasonal influenza vaccine for multiple sclerosis patients in treatment with ocrelizumab: an expert consensus

**DOI:** 10.1007/s00415-021-10466-0

**Published:** 2021-02-20

**Authors:** Massimo Filippi, Ruggero Capra, Diego Centonze, Claudio Gasperini, Francesco Patti, Paola Perini, Carlo Pozzilli, Maria A. Rocca, Antonio Uccelli, Maria Trojano

**Affiliations:** 1 Neurology Unit, MS Center, IRCCS San Raffaele Scientific Institute, Via Olgettina, 60, 20132 Milan, Italy; 2 Neurorehabilitation Unit, IRCCS San Raffaele Scientific Institute, Milan, Italy; 3 Neurophysiology Service, IRCCS San Raffaele Scientific Institute, Milan, Italy; 4 Neuroimaging Research Unit, Division of Neuroscience, IRCCS San Raffaele Scientific Institute, Milan, Italy; 5 Vita-Salute San Raffaele University, Milan, Italy; 6 Multiple Sclerosis Center, Spedali Civili of Brescia, Montichiari, Brescia Italy; 7 Department of Systems Medicine, Tor Vergata University, Rome, Italy; 8 Unit of Neurology, IRCCS Neuromed, Pozzilli, IS Italy; 9 Department of Neurosciences, San Camillo Forlanini Hospital, Rome, Italy; 10 Department of Medical and Surgical Sciences and Advanced Technologies, GF Ingrassia, University of Catania, Catania, Italy; 11 Multiple Sclerosis Center, PO G Rodolico, University of Catania, Catania, Italy; 12 Multiple Sclerosis Centre of the Veneto Region, Neurology Clinic, Department of Neurosciences (DNS), University of Padua, Padua, Italy; 13 Multiple Sclerosis Center, ‘S. Andrea’ Hospital, Rome, Italy; 14 Department of Neurology, Rehabilitation, Ophthalmology, Genetics, Maternal and Child Health (DINOGMI), University of Genoa, Genoa, Italy; 15 Neurology and Neurophysiopathology Unit, Department of Basic Medical Sciences, Neuroscience and Sense Organs, University of Bari “Aldo Moro”, Bari, Italy

Dear Sirs,

The severe acute respiratory syndrome coronavirus 2 (SARS-CoV-2) causing coronavirus disease 2019 (COVID-19) has killed hundreds of thousands of humans in a global pandemic and severe COVID-19 is often associated with lymphopenia, initially causing great concern over the use of immunosuppressive agents. In some cases, this led to the cessation or delay of treatment of autoimmunity. However, it is increasingly evident that lymphopenia is a consequence rather than a cause of infection. While the immune system eliminates SARS-CoV-2 in most individuals, viral escape, immune exhaustion and elevated cytokine release can lead to hyperactivation of the innate immune response, vascular damage and hypercoagulation, which can lead to significant morbidity, acute respiratory distress, multi-organ failure and, in some cases, death. Immunotherapy may have some value in treating severe COVID-19 [1], but considering specifically MS and all currently available disease modifying treatments (DMTs) it is important to understand how they influence susceptibility to infection and length of the carrier state, and to consider how DMTs may influence immunity to reinfection and potential vaccine responses. Besides these considerations, it appears that the innate immune response [2], and perhaps later anti-viral CD8 T-cell responses, could eliminate the SARS-CoV2 before significant antibody responses have developed, suggesting that most MS treatments, which largely exhibit limited persistent effects on the innate immune and CD8 T-cell responses, would have limited influence on COVID-19. SARS-CoV-2 is eliminated by the majority of people with MS and other autoimmune conditions on immunotherapies without significant consequences. Anti-viral antibodies neutralize the virus and can contribute to the elimination of the primary SARS-CoV2 infection. However, B cells do not appear to be an absolute requirement for recovery. This is shown by the recovery of people genetically lacking B cells, such as with X-linked hypogammaglobulinaemia [3], and is reinforced by the fact that the majority of MS patients treated with CD20 B-cell depleting agents recover from COVID-19 [4, 5]. Ocrelizumab was also reported not to influence the generation of effective T and B response to primary varicella zoster virus infection [6]. Furthermore, B-cell depletion is unlikely to influence, or be significantly influenced by, the vascular pathology and hypercoagulopathy that are major pathological features in COVID-19.

Nonetheless some publications have recently raised some concerns regarding the use of an anti-CD20 in a 6-month schedule, like ocrelizumab, and some of the points coming up from these data forced an analysis and a discussion among clinicians, expert in the diagnosis and treatment of MS, in Italy. Their review of the data available until November 4^th^ brought to some joint agreements:

1. Ocrelizumab, as a B-cell depleting therapy, seems to minimally impact the innate immune system particularly needed in neoantigen and anti-viral response [7–9]. In the ocrelizumab all-exposure population, the rate of serious infections remained low over 7 years of treatment and was consistent with rates of infection-related hospitalizations reported in real-world MS cohorts [10];
2. People with MS appear to respond to SARS‐CoV‐2 in a similar way to the general population, where severe disease is notably influenced by age and comorbidities, such as diabetes and obesity;
3. Risk factors for a severe COVID-19 disease in people with MS are high disability or a progressive course of the disease [11];
4. Available data still do not allow to conclude that the risk of acquiring COVID-19 depends upon the duration of treatment with ocrelizumab [12, 13];
5. Some evidence suggests that patients treated with ocrelizumab are more likely to be hospitalized with COVID-19 and need intensive care versus patients treated with other DMTs [11, 13, 14], but case-fatality rates in patients with MS receiving ocrelizumab appear to be in line with those in MS patients and the general population [15].

Therefore, considering that SARS-CoV-2 does not seem to significantly impact this neuroimmunological disease, which if not treated can lead to a poor long-term prognosis and persistent disability, especially when very active [12], and that experiences coming from the first pandemic wave have not brought to a different risk stratification in terms of DMTs, we suggest to maintain pre-pandemic criteria in the therapeutic choice [16].

Vaccination against communicable diseases is part of general health maintenance and an important aspect of multiple sclerosis (MS) disease management because infections can exacerbate MS symptoms and are a recognized complication of some MS treatments [17]. Live vaccines are generally contraindicated during DMTs [18], furthermore some data suggest that immunosuppressive DMTs may reduce the effectiveness of inactivated vaccines, such as that for seasonal influenza [18].

Seasonal influenza epidemics, on the other hand, can cause significant illness, hospitalization and death during Autumn and Winter. While influenza infection is usually mild and uncomplicated, it can cause severe outcomes in some specific cases such as among the elderly, people living in health care facilities and other vulnerable groups, including persons with underlying medical conditions. In particular, co-circulation of influenza viruses during the ongoing COVID-19 pandemic in Autumn and Winter might have severe consequences for vulnerable populations and place and additional burden on health systems already strained by COVID-19 [19].

For all these reasons the joint MS expert panel, in line with WHO, supports the following statements regarding the administration of seasonal influenza vaccine to MS patients treated with ocrelizumab:

- All MS patients should be vaccinated (with inactivated vaccines), especially the most vulnerable ones, i.e., patients with progressive forms of MS, who are usually characterized by risk factors such as age, disability and comorbidities;
- Vaccine and treatment recommendations for 2020–2021 are substantially in line with previous years; in particular, for MS patients treated with ocrelizumab who plan to receive seasonal influenza vaccination no changes are foreseen:
  o especially this year, the administration of the inactive seasonal influenza vaccine is to be considered for the general population; respiratory comorbidities are considered risk factors for COVID-19 severity [4, 5] and a potentially overlap of seasonal influenza and SARS-CoV-2 infection could determine the worst scenario in some cases;
- Phase III studies demonstrate that ocrelizumab-treated patients maintained the preexisting specific humoral immunity to common viral and bacterial agents achieved before treatment initiation [17];
- In the Veloce trial, a randomized open-label study, relapsing–remitting MS patients showed humoral responses, although decreased, to a neoantigen and to some clinical relevant vaccines, including the seasonal influenza vaccine. For this reason, it is recommended to vaccinate MS patients treated with ocrelizumab with inactivated seasonal influenza vaccines [9, 17];
- Regarding timing of vaccination before starting ocrelizumab and during the treatment, physicians should follow the available Summary of Product Characteristics (SmPC) and common sense; peripheral B-cell depletion, in fact, does not mirror the same degree of depletion in secondary lymphoid organs [20]; for this reason the suggested timing is:
  o in patients being considered for treatment with ocrelizumab, vaccination should be administered and completed ≥ 2 weeks before the first administration [18];
  p in patients already receiving ocrelizumab, vaccinations should not overlap the infusion and, in the absence of an immunological rationale, a 3-months’ delay is proposed, in order to have a potentially protective humoral response to the vaccine even if attenuated.

It is absolutely clear that more data are needed to deeply understand the humoral protection efficacy and the success of the vaccination strategy in a larger pool of patients.
